# Spatial distribution characteristics and accessibility analysis of beautiful leisure villages in China

**DOI:** 10.1371/journal.pone.0276175

**Published:** 2022-10-26

**Authors:** Zhenjie Liao, Lijuan Zhang, Xuanfei Wang

**Affiliations:** 1 School of Management, Guangzhou Huashang College, Guangzhou, China; 2 School of Information, Guangdong University of Finance & Economics, Guangzhou, China; Northeastern University (Shenyang China), CHINA

## Abstract

This study was designed to evaluate the spatial distribution characteristics of 1432 beautiful leisure villages in China using econometric geography and spatial geographic information system analysis methods, such as nearest distance index, K index, and nuclear density. We also used the grid cost weighted distance algorithm to determine the spatial accessibility of beautiful leisure villages and the overall accessibility of county units. In addition, our evaluations determined the spatial differences in county accessibility using exploratory spatial data analysis (ESDA). Our results showed that the spatial distribution of the beautiful leisure villages in China could be best described using the cohesion type classification and that there were large differences in their distribution between provinces and economic regions. The average accessibility time of beautiful leisure villages was 197.24 min with only 57.19% of these commutes being less than 2 h, and only 17.88% being less than 30 min. The area with the longest accessibility time was located on the Qinghai Tibet Plateau, at up to 1510.03 min. The spatial distribution of accessibility showed obvious traffic directivity producing a positive Moran I value for most counties. There was also a significant positive correlation between the accessibility of beautiful leisure villages and their adjacent areas, and clear patterns of hot spots–sub-hot spots–sub-cold spots—cold spots from east to west. The overall service scope of beautiful leisure villages was characterized by west > east > middle, with topography, population, economy, and location acting as the major factors in the spatial distribution of these beautiful leisure villages in China.

## 1. Introduction

Rural areas shoulder a responsibility in promoting the modernization of grassroots governance and building a healthy society in a sustainable manner. However, the rapid development of both the economy and society and the widespread promotion of modernization, rural emptying, and poverty are becoming increasingly serious problems. Some villages commonly demolish houses, destroy fields, disrupt the area’s ecology, and pollute the environment in an effort to develop local industry. In some areas with particularly poor locations and extremely limited resources, weak development foundations result in the gradual decline of these villages and in some extreme cases, their dying out [1]. Efficient, high-quality development of the agriculture sector and the harmonization of industrial and sustainability requirements help to make the countryside a beautiful alternative for farmers and their families and meet the leisure tourism consumption needs of urban and rural residents. Accordingly, the Ministry of Rural Agriculture selected a number of beautiful leisure villages across China, all of which presented strong agricultural value, unique ecology, and sustainable farming for further development, starting in 2014. These sites have been referred to as “beautiful leisure villages” for the majority of this text. Under this context, we designed this study to assess these developments as a research object to evaluate how spatial distribution and various other influencing factors might be conducive to optimizing the spatial layout of beautiful leisure villages that can promote the development of regional urban–rural integration and promote the rural revitalization strategy.

The construction of beautiful leisure villages is an important part of the rural revitalization strategy and has attracted the attention of researchers worldwide. Related research on villages with tourism and leisure functions has mostly adopted questionnaire surveys, model construction, and case analysis, focusing on the topics of social management [2], emotional behavior [3], site selection [4], and sustainable development [5]. The earliest research on these villages in China began in Taiwan. With the in-depth promotion of new urbanization in mainland China and the complete implementation of rural revitalization strategies, leisure or tourism villages have become a research hotspot. This research is focused on case analysis and problem solving, social space restoration and reconstruction, and sustainable development. Of these topics, research into the geospatial structure, using agritainment [6], homestays [7], and rural tourist destinations (scenic spots) [8] as the major characteristics, has had the most significant impact on the micro and meso scales. However, despite some research focusing on regional units that allow for the evaluation of these beautiful leisure villages at the macro scale [9, 10], research on the influencing factors for these sites remains scarce and does not include evaluations of different types of villages, consequently, leaving a gap in the guidance for their selection and development [11]. This study seeks to create a framework to more realistically evaluate the macro context of these sites and lays a foundation for further understanding of this research and the improvement of the methods used during evaluation.

Previous researchers studied the spatial structure of tourism, mainly using location theory, central periphery theory, core edge model, and other relevant theoretical models, to explore the source market of tourism destinations [11], and spatial structure and its evolution process [12, 13]. Moreover, previous studies mainly involved A-level scenic spots [14], wetland parks [15], characteristic towns [16], but research on the spatial structure of beautiful leisure villages is scarce. To further expand the existing research on the spatial pattern of beautiful leisure villages, spatial distribution and accessibility distribution patterns of beautiful leisure villages can be combined that can provide theoretical support for the planning and layout of such villages. Spatial accessibility represents the basic law of human activities, which refers to the ability to arrive at a designated place at an appropriate time through different transportation facilities. It depends on human mobility and the opportunity to reach the desired destination through mobility. Ever since Hansen first proposed the concept of accessibility in 1959 [17], several scholars have conducted in-depth research on accessibility, mainly on the layout and location of social public service facilities [18, 19], relationship between road network and accessibility in different regions, and factors affecting accessibility [20, 21]. Moreover, the research areas have been relatively small, for example, schools, parks, and hospitals [22]. The research methods mainly included mobile search method, 2SFCA, gravity method, and gravity model [23]. However, considering the different levels of highway and railway networks, geographic information technology (GIS) grid cost-weighted distance has rarely been used to calculate the accessibility of beautiful leisure villages. Thus, from a macro-perspective, this study integrated mathematical statistical analysis methods and modern GIS with traditional geospatial analysis methods and spatial measurement methods, to analyze the spatial distribution characteristics of 1432 beautiful leisure villages in China. Further, GIS grid cost-weighted distance was used to calculate the accessibility of these villages based on different levels of highway and railway networks, identify the degree of influence of different factors on the evolution of the spatial patterns of the villages, and comprehensively analyze the spatial heterogeneity of the influencing factors of the villages, to reveal the internal evolution mechanisms of the spatial patterns of the villages, and provide theoretical support and practical reference to optimize the spatial layout and policy formulation of beautiful villages in the future. The study is expected to provide practical guidance for future rural construction and development.

## 2. Methods and materials

### 2.1. Data sources

Since their first inception by the Ministry for Rural Agriculture of China in 2014, an additional 1432 beautiful leisure villages (excluding Hong Kong, Macao, and Taiwan) have been selected for adaptation over the course of eight batches concluding at the end of 2021. We obtained a complete list of these villages from the official Ministry website (http://www.moa.gov.cn/) (as shown in list of beautiful leisure villages in China) and then used Google Maps to determine their spatial locations via calibration with the existing detailed maps of all provinces and cities in China. This research used all the counties and cities above the prefecture level after the 2020 merger as research units (excluding Taiwan Province, Hong Kong, and Macao SAR), resulting in 2856 villages in total for analysis. The spatial administrative boundary vector data were obtained from the 1:4000000 basic geography information database of China, and traffic networks were used to create the basis for realizing spatial accessibility. All road data were obtained from the vectorization of the national 1:4500000-traffic map by the National Map Publishing House in 2020. Notably, the reachability calculated in this study is only a theoretical value and does not consider any extreme conditions (such as the impact of traffic congestion and the combination of different traffic modes). We used ArcGIS 10.6 software to project all the graphic data onto the Albert equal-product conic projection system, and the basic geographic information data were hierarchically vectorized and stored within the geographic database. The statistical data were acquired from the “China Statistical Yearbook” and the statistical yearbooks of all provinces and municipalities that are directly under the central government and autonomous regions. Moreover, spatial distribution patterns, spatial distribution balance, spatial distribution density, spatial autocorrelation and accessibility, and other indicators were used to explore the spatial distribution characteristics of the selected beautiful leisure villages in China.

### 2.2. PPA method

Point pattern analysis is commonly used to reveal the spatial distribution of research objects by evaluating the spatial location of point events. Usually, the nearest neighbor index (NNI) method, which is based on distance, and the kernel density estimation (KDE) method, which is based on density, are used to analyze the spatial distribution of research point entities from two aspects: dispersion and aggregation.

#### 2.2.1. NNI

NNI compares the deviation of specific distributions from the random distribution by calculating the ratio of the average distance of the nearest neighbor pair to the average distance in the random distribution model as follows [24]:

$$NNI=\frac{\left[\sum_{i=1}^{N}\frac{\text{min}(d_{ij})}{N}\right]}{0.5Sqrt(A/N)},$$ (1)

where min(*d*_*ij*_) is the distance between any point and its nearest neighbor, n is the total number of beautiful leisure villages, and a is the study area.

#### 2.2.2. KED nuclear density equation

The KED nuclear density equation can be described as follows [25]:

$$f_{n}(x)=\frac{1}{nh^{d}}\sum_{i=1}^{n}k(\frac{x-X_{i}}{h}),$$ (2)

where *K(x-x*_*i*_*/h)* is the kernel density equation, *x*_*i*_ is the kernel density of each point, *x* is the kernel density at the center of the grid, *h* is the threshold, *n* is the number of points within the threshold range, and D is the dimension of the data. Its geometric function requires that the density at the center of each *x*_*i*_ point is the highest and that this decreases as the points move away from the center. When the distance from the center reaches a certain threshold value (the edge of the window), the density is zero, and the core density at the grid center *x* is the sum of the densities within the threshold range.

#### 2.2.3. K index

The *K* index was introduced to analyze the spatial distribution of beautiful leisure villages at different scales. This value is calculated as follows [26]:

$$K(t_{s})=\frac{A}{H^{2}}\sum_{i}^{}\sum_{}^{i\neq j}I(t_{ij}),$$ (3)

where *I(t*_*ij*_*)* is a beautiful leisure village in a circle with I as the center and *t*_*s*_ as the radius, excluding *i* itself. *K(t*_*s*_*)* can be transformed into its square root *L(t*_*s*_*)*:

$$L(t_{s})=SQRT\left[\frac{K(t_{s})}{\pi }\right]-t_{s},$$ (4)

where an *L(t*_*s*_*)* >0 reveals an aggregated distribution, *L(t*_*s*_*)* <0 reveals a diffused distribution, and *L(t*_*s*_*)* = 0 represents a completely random distribution.

### 2.3. Equilibrium index

#### 2.3.1 Geographical concentration index

This index is used to measure the concentration of beautiful leisure villages within specific provinces and is calculated as follows [27]:

$$E=100\times \sqrt{\sum_{i=1}^{n}\left(P_{i}/S\right)},$$ (5)

where *E* is the geographical concentration index, *P*_*i*_ is the number of beautiful leisure villages in *province i*, *S* is the total number of beautiful leisure villages, and *n* is the number of provincial and urban areas (n = 31). The greater the *E* value, the more concentrated the distribution of the beautiful leisure villages. If beautiful leisure villages are evenly distributed in all provinces and urban areas, their concentration index is:

$$\bar{E}=100\times \sqrt{\sum_{i=1}^{n}{\left(1/n\right)}^{2}}=17.96,$$

#### 2.3.2. Gini coefficient

This value was used to measure the distribution of beautiful leisure villages among each of the regions of China and was calculated as follows [28]:

$$Gini=-\sum_{i=1}^{m}\left(D_{i}\text{ln}\;\text{D}i\right)/\text{ln}m,$$ (6)

where *Gini* is the Gini coefficient, *D*_*i*_ is the percentage of beautiful leisure villages in the corresponding study area as a portion of the national total, and *m* is the number of study areas. The larger the *Gini* coefficient, the higher the concentration of beautiful leisure villages in the region.

#### 2.3.3. Imbalance index

This value was used to evaluate the completeness of the distribution evaluations at various scales and was calculated as follows [29]:

$$B=(\sum_{i=1}^{n}C_{i}-50(n+1))/(100n-50(n+1)),$$ (7)

where *B* is the imbalance index and *C*_*i*_ is the proportion of beautiful leisure villages in each research unit as part of the total sample number. The proportion of each research unit was then ranked from large to small and this was used to calculate the cumulative percentage, Ci, of the ranked *i*, and *n* is the number of research units. *B* = 0 where beautiful leisure villages were evenly distributed at all levels and *B* = 1 where there is an extreme imbalance at a specific scale.

### 2.4. Accessibility evaluations

#### 2.4.1. Cumulative consumption distance

Spatial accessibility is measured using the cumulative consumption distance method, which uses a grid as the basic operation unit and calculates the shortest weighted distance from each grid to a destination grid using the shortest path method. These values are calculated as follows [30]:

$$F=\left\{\begin{array}{l} \frac{1}{2}\sum_{i=1}^{n}\left(C_{i}+C_{i+1}\right)\\ \frac{\sqrt{2}}{2}\sum_{i=1}^{n}\left(C_{i}+C_{i+1}\right) \end{array}\right.,$$ (8)

where *C*_*i*_ is the consumption value of *pixel i*, *C*_*i+1*_ is the consumption value of pixel i, and *n* is the number of pixels. If these values are calculated in a vertical or parallel direction across the cost surface, the upper fraction of Eq (8) is adopted; if the distance is calculated diagonally across the cost surface, the lower fraction is used.

The political districts of China were rasterized using a grid size of 1 km × 1 km. Further, we used the technical standards provided in the highway engineering manual of the People’s Republic of China (JTGB01-2003) and the quality of road networks at different times to assign different time cost values to each grid as described by the standards summarized in Table 1. When we considered the availability of data, we limited our evaluations to road accessibility and replaced other modes of access with a walking speed of 5 km·h-1. The grid through which the water system passes was set as the barrier grid, and its value was null. Thus, once the vector elements were extracted from the basic database and given cost values, they were converted into grid data, and the time-cost-value grid diagrams of different road layers were superimposed to produce a single global time-cost grid diagram.

**Table 1:** Velocity and cost of the cantonal land traffic network.

Road type	Speed(km·h^-1^)	Time cost(min)
High speed train	250	0.24
Railway	120	0.5
Expressway	120	0.5
National Highway	90	0.67
Provincial Highway	80	0.75
County Highway	60	1
Other	20	3

#### 2.4.2. Overall accessibility of county-level units

We then described the convenience of the daily arrival of regional residents to these beautiful leisure villages as a whole to reflect the spatial structure of accessibility to these sites at the regional level. Given this, we introduced a novel factor, the overall accessibility of county-level units, which is designed to reflect the accessibility of the entire administrative unit by calculating the average value of grid accessibility for each administrative unit. This was calculated as follows [31]:

$$K_{j}=\sum_{i=1}^{n_{j}}F_{i}/n_{j},$$ (9)

where *K*_*j*_ is the accessibility of the beautiful leisure village of *administrative unit j*. The smaller the *K*_*j*_, the more convenient it is for the administrative unit to reach the beautiful leisure village; *n*_*j*_ is the number of grids within the scope of the *j* administrative unit; and *F*_*i*_ is the accessibility time of the *ith* grid [32].

### 2.5. ESDA spatial correlation analysis

The global Moran’s I index of the ESDA was used to evaluate and identify the spatial correlation structure patterns for these villages. If Moran’s I is significantly positive, it implies that the regions with high or low accessibility are significantly concentrated in space, while exhibiting significant spatial differences in the accessibility between the region and its surrounding areas. Getis-Ord Gi* is used to identify high-value and low-value clusters at different spatial locations and we standardized Gi*(d) to Z(Gi*) to facilitate more meaningful comparisons [33]. Thus, if Z(Gi*) is positive and significant, it indicates that the value around location i is relatively high and that it belongs to high-value spatial aggregation (hot spot). Conversely, if Z(Gi*) is negative and significant, it indicates that the value around position i is relatively low and belongs to a low-value spatial aggregation (cold spot).

## 3. Results and discussion

### 3.1 Space release type

The beautiful leisure villages evaluated in this study are distributed across 31 provinces (cities and districts) but demonstrate uneven distribution characteristics. The province with the largest number of villages is Zhejiang, (70), while Ningxia has the least number of villages (22). On an average, each province has 46.2 beautiful leisure villages, with the highest density observed in Shanghai, at 53.97 per 10000 km2, and the lowest in Tibet. The average density of beautiful leisure villages in China is 1.49 per 10000 km^2^.

**Fig 1:**
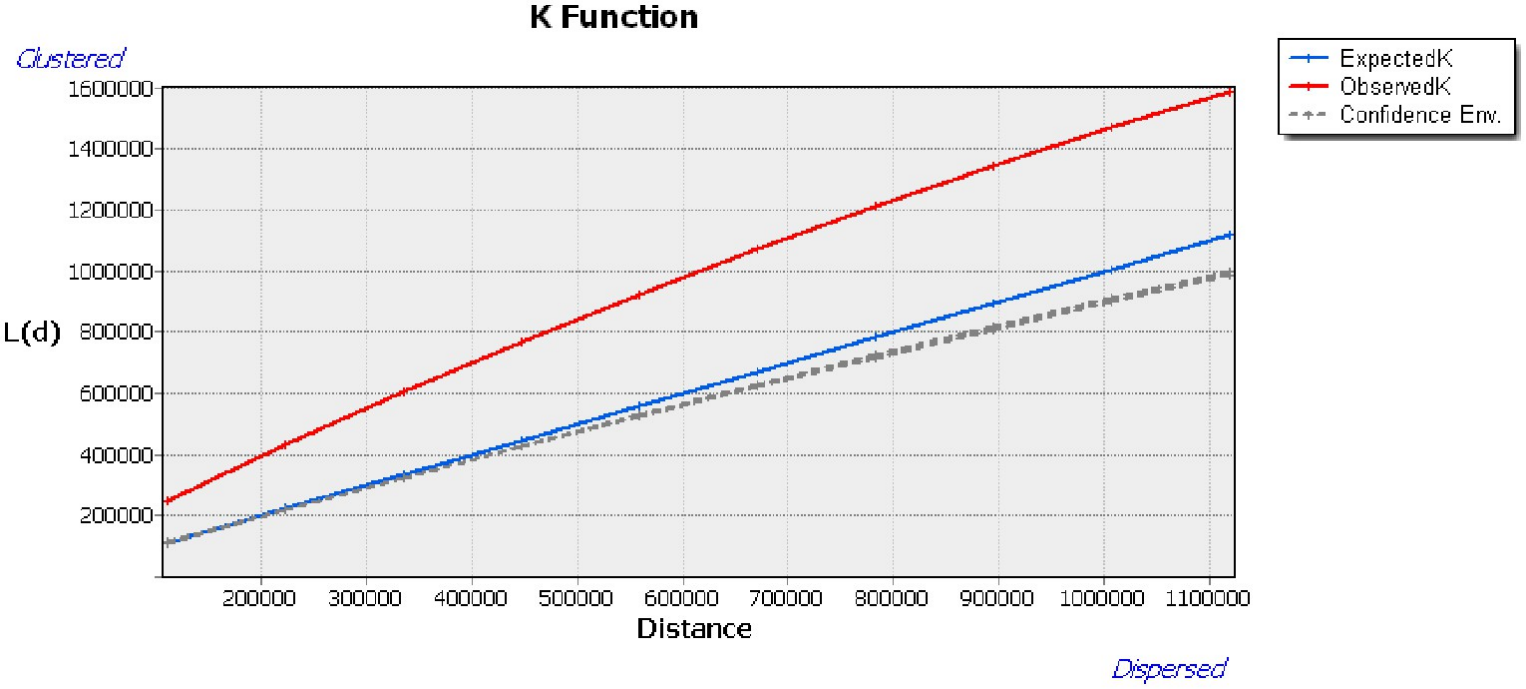
Results of Ripley *K(r)* function for beautiful leisure villages.

China has successively implemented eight batches of beautiful leisure village developments, which were all completed between 2014 and 2021. The NNI for each batch of the beautiful leisure villages was calculated using ArcGIS software, which determined that the NNI of the beautiful leisure villages developed over this period was less than 1, indicating that their spatial distribution was likely to appear aggregated (Table 2). We then used Crimestat software to evaluate the agglomeration of these beautiful leisure villages via the Ripley *K(r)* function (Fig 1), which revealed that the agglomeration of these developments was significantly higher than the maximum value for random distribution, indicating that within the scale of this study, beautiful leisure villages present as an agglomeration, with their aggregation initially increasing and then decreasing with increasing distance.

**Table 2:** Nearest neighbor distance index of beautiful leisure villages distribution.

Batch	Year	Average nearest distance(km)	Expected nearest distance(km)	NNI
1	2014	128.910	158.779	0.812
2	2015	103.385	144.945	0.713
3	2016	107.425	129.643	0.829
4	2017	111.610	129.643	0.861
5	2018	107.729	129.643	0.831
6	2019	85.103	98.471	0.864
7	2020	93.427	100.825	0.927
8	2021	86.945	99.627	0.873

### 3.2 Spatial distribution balance

Table 3 shows the results of the geographical concentration index, Gini coefficient, and imbalance index for each batch of beautiful leisure villages. The results show that the geographical concentration index of the eight groups of beautiful leisure villages was greater than the maximum average distribution value (18.53). This indicated that since the establishment of these projects in 2014, the spatial distribution of these developments has been largely imbalanced at the provincial level, with most villages concentrated in specific parts of the country. However, this imbalance tends to weaken gradually. The traditional zoning of China’s geographical regions divides the country into seven regions: northern, northeastern, eastern, central south, southern, southwestern, and northwestern China. The number of beautiful leisure villages in each region for each batch was counted, and the Gini coefficient was calculated to determine the balance of beautiful leisure village distribution across the country (Table 3). This analysis revealed that the Gini coefficient of these eight groups of beautiful leisure villages fluctuated from 0.930 to 0.965, suggesting that the spatial distribution of these developments has always been slightly imbalanced when evaluations were conducted at the cross-provincial economic zone level, and that there is a trend towards centralized distribution in this data.

**Table 3:** Geographic concentrate index, Gini coefficient and unbalanced index of beautiful leisure villages distribution.

Batch	Year	E	Gini	B
1	2014	19.079	0.952	0.168
2	2015	19.039	0.952	0.165
3	2016	18.595	0.962	0.144
4	2017	18.499	0.965	0.142
5	2018	18.571	0.963	0.146
6	2019	18.748	0.957	0.107
7	2020	19.041	0.955	0.200
8	2021	18.988	0.930	0.196

The imbalance index, E, of each batch of beautiful leisure villages was calculated (Table 3) to determine the completeness of the distribution at each scale. This analysis revealed that the imbalance index for the eight groups of beautiful leisure villages fluctuated from 0.107 to 0.200, indicating that the spatial distribution of these developments at the provincial scale has been mostly incomplete; additionally a trend toward centralized distribution was observed. These evaluations revealed a bias towards the east and middle sections of the country with the highest population and industry clusters, especially in Shandong, Hunan, Heilongjiang, Hubei, Jiangxi, Inner Mongolia, Zhejiang, Jiangsu, Anhui, and Henan, where the annual number of approved villages is approximately 50% of the total villages.

### 3.3 Spatially distributed nuclear density

KDE was conducted to visually detect hot spot areas within the centroid data of these beautiful leisure villages. The search range adopted the default value and the output was a 1 km × 1 km grid, which confirmed that the distribution of these beautiful leisure villages across China demonstrated clear spatial agglomeration. There were six high-density hot spots: eastern Hubei, northern Hunan, southern Jiangsu-northern Zhejiang, southwestern Shandong, northern Ningxia, and central and southern Heilongjiang. Medium-density sub-hot spots primarily included central Hubei, Sichuan Chongqing junction, Guanzhong, Jinzhong, northern Henan, central Liaoning, southern Jiangxi, Hunan Jiangxi Hubei junction, and eastern Shandong.

### 3.4 Spatial pattern of accessibility

#### 3.4.1 Overall spatial distribution characteristics

**Fig 2:**
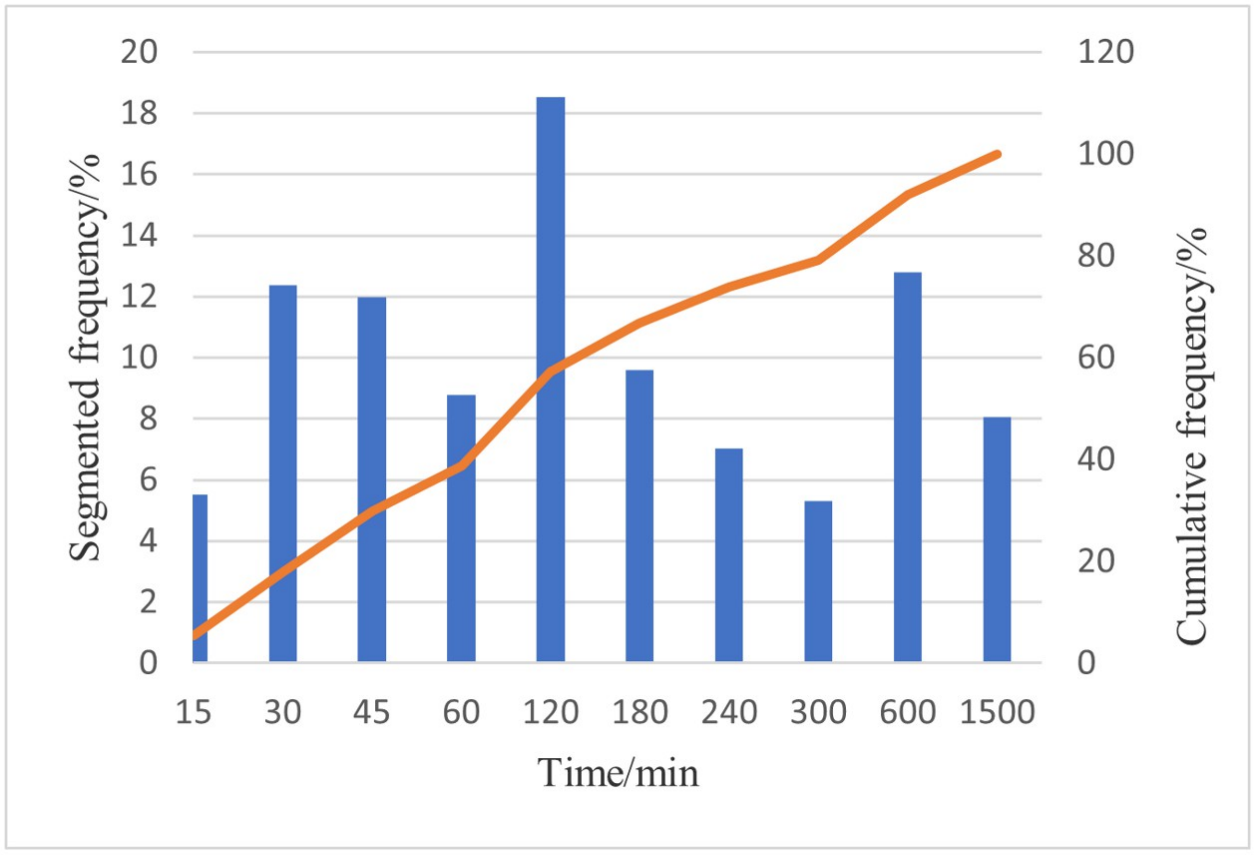
Time of spatial accessibility of beautiful leisure villages.

Considering the beautiful leisure villages as the source points of consumption distance, we calculated the time spent traveling to each beautiful leisure village using the traffic network from any grid within the central land mass of China, which can be determined through traffic line reversal. The accessibility of this grid to each beautiful leisure village was determined using the minimum time spent to reach each development from any point within the transportation network across China. Subsequently, considering 15, 30, and 45 min, and 1, 1.5, 2, 3, 5, and 8 h as the standards, the accessibility of each of these developments was divided into 10 periods. We then calculated the spatial distribution frequency and cumulative frequency of these 10 periods (Fig 2), and evaluated the relationship between the areas occupied by each period. The average accessibility time for these beautiful leisure villages was 197.24 min. Further, approximately 57.19% of the beautiful leisure villages were accessible within 2 h, with the highest accessibility areas, while those within 30 min of a major node accounted for only 17.88% of the total area, and the low accessible areas, which were > 5 h from the closest node accounted for 20.87% of the total area. The spatial distribution frequency for each period revealed that the 1–2 h group was the most widely distributed, accounting for 18.53% of the total area of the country, followed by the 45–60 min group, which accounted for 12.37%. Few areas had relatively poor accessibility to the beautiful leisure villages, and the proportion of areas with > a 10-h commute remained high at 8.05%. The differences in the distribution and accessibility of these areas across the country were evident, with the regions on the Qinghai Tibet Plateau, Tarim Basin, Junggar Basin, Alxa, northern Inner Mongolia, Western Sichuan, Daxinganling, Southeast Tibet, and other mostly alpine, desert, ethnic minority, or border areas experiencing the worst accessibility, with some regions having a minimum accessibility time of 1510.03 min. The regions with good accessibility were primarily distributed across the Central Plains, Yangtze River Delta, Pearl River Delta, Bohai rim, Liaoshen, and other central and eastern regions, while the regions with good accessibility in the West showed obvious traffic directivity, mainly along the traffic trunk lines, such as Baolan, Longhai, Nanjiang, Lanxin, Baocheng, Chengdu Chongqing, and Guiyang Kunming.

#### 3.4.2 Spatial differences in county unit accessibility

This study was designed to evaluate the overall accessibility differences and regional effects of beautiful leisure villages from a macro perspective. Accordingly, we divided the regions of the entire country into small areas, using the county level for evaluation, as it acts as the most basic regional economic unit in China; moreover, most of China uses county-level administrative units for regional division. Division at this level implied that some municipal areas of some cities were merged. Thus, our county-level unit division produced 2596 areas for evaluation. The accessibility of the entire beautiful leisure countryside was calculated using Eq (9), and the accessibility of each county unit was divided into five levels using the natural fracture classification method. The combination of the accessibility characteristics of specific counties with different time periods allowed us to determine the overall accessibility characteristics of China’s beautiful leisure villages, which can be defined as follows: Most high-accessibility counties, with accessibility of < 44.83 min, are scattered but show a slight bias towards the Central Plains, Huang Huai, Hubei, Yangtze River Delta, and other regions. The next highest accessibility counties, with accessibility of 44.83–108.40 min, were generally distributed towards the east of the Erlianhot Luxi line but protrude into the Hexi Corridor in the Ningxia Longdong area. The area to the west of this line was only sparsely populated with these developments with one or two dotted across some areas of southern Tibet and Northern Xinjiang. The number of medium accessibility counties with accessibility of 108.40~237.63 min was small, generally scattered in patches and blocks, and arranged outside the sub-high-accessibility counties, or filling in the blanks within the interior regions. These areas were only distributed in strips east of Qinghai, north of Xinjiang, the big and small Xing’an Mountains, and the junction of Yunnan and Guizhou. The sub-low accessibility counties with accessibility of 237.63~512.00 min showed a relatively continuous decentralized block pattern and were primarily clustered in a ring on the outer edge of the Qinghai Tibet Plateau and northern Inner Mongolia. All counties with accessibility of > 512.00 min fell within the intersection of Qiangtang in northern Tibet, Tanggula in Qinghai, and southern Xinjiang, forming a huge patch in this area.

### 3.4.3 Spatial relevance of accessibility distribution

*ESDA* spatial correlation analysis allowed us to explore the spatial distribution pattern of this accessibility index. These evaluations revealed that the Moran’s I value of China’s beautiful leisure villages was 0.13, and the Z value was 40.19. These test results were significant, indicating that there is a strong positive spatial correlation between counties, thus, showing a strong agglomeration pattern. We then studied the location and regional correlation between accessibility and aggregation using the local spatial correlation index Getis-Ord Gi* calculated and spatialized using ArcGIS software. The local Gi* statistics were divided into five categories from high to low using the Jenks natural fracture method, producing hot spot, sub-hot spot, middle, sub-cold spot, and cold spot areas. This data was then used to generate a hot spot map of the accessibility and spatial distribution of these beautiful leisure villages at the county level. The visualization revealed that the spatial correlation, degree of spatial aggregation, and accessibility of the county unit level were more significant than those anticipated initially following the data. This distribution presents an obvious pattern of sub-hot spot–sub-cold spot zonal distribution from east to west with the spatial distribution of the hot spots being the most fragmented. This also revealed that the secondary hot spots were most widely distributed across the eastern and central regions, while the cold spot areas were concentrated near Qinghai Tibet and Southern Xinjiang, with the sub-cold spot areas distributed around the periphery of these cold spots.

### 3.5 Service scope measurement

The service scope of each beautiful leisure village was estimated based on their spatial accessibility. This division standard implied that, if the time from any grid in the study area to a specific beautiful leisure village was less than that from the grid to any other beautiful leisure village, this grid fell into the service scope of this development. By analogy, the service scope of I is a region composed of a series of continuous grids. The grid at the boundary of the service scope would have equal accessibility to two beautiful leisure villages. Given this principle, we could divide 1432 beautiful leisure villages into their respective service ranges. The analysis of these service ranges revealed that the service scope for each village could be characterized as central > eastern > western, because most beautiful leisure villages were concentrated in the central region, with its prominent rural resource endowment, and the economically developed areas in the east. There was a distinct lack of beautiful leisure villages in the west, especially in the arid areas of northwest China. Comparisons with the accessibility map demonstrated that the service scope of areas with poor spatial accessibility was generally large, while the service scope of beautiful leisure villages in Central China, where there were dense beautiful leisure villages, was small. Thus, there were significant differences in the service scopes of various beautiful leisure villages. The largest service scopes belonged to Kaima Village, Luoma Town, Seni District, Naqu City, Tibet Autonomous Region, with an area of 39.76×104 km2, followed by Xuechong village, Dongga Township, Sangzhuzi District, Shigatse City, Tibet Autonomous Region, with an area of 37.92×104 km2.

### 3.6 Evaluating the factors influencing the spatial distribution of beautiful leisure villages across China

The spatial distribution of China’s beautiful leisure villages was restricted and influenced by various factors to different extents. Based on the results of previous relevant studies and considering the scientificity and availability of data comprehensively, the terrain factor, population factor, economic factor, location factor and other influencing factors were finally selected for research. Among them, terrain factors included altitude and slope. The above four factors were selected for analysis, and the corresponding results are shown in Table 4. Terrain and economic factors were significantly correlated with the spatial distribution of the beautiful leisure villages. Similarly, the population factor was strongly correlated to the spatial distribution of the beautiful leisure villages. Contrastingly, location factors and the spatial distribution of beautiful leisure villages were weakly correlated. Thus, terrain, economic, and population factors represented major influencing factors, while the location factor represented a secondary factor.

**Table 4:** Correlation between spatial distribution and geographic space of beautiful leisure villages in China.

Influencing factor	Index factor	Spearman correlation coefficient	significance(two-tailed)
Topographic factors	Elevation and Slope	-0.912**	0.000
Demographic factors	Regional population	0.607**	0.000
Economic factors	Per capita GDP	0.693**	0.000
Location factors	Distance from the city	-0.577**	0.001

**,* represented significant correlation at 0.01 and 0.05 levels respectively

#### 3.6.1 Topographic factors

China’s terrain shows high elevation in the west and low elevation in the east, while beautiful leisure villages were generally more common in the southeastern part of China and less common in the northwest regions. Most beautiful leisure villages were distributed in areas with low elevations and low slopes, indicating a strong inverse relationship between the distribution of beautiful leisure villages and slope gradient. The number of beautiful leisure villages decreased with the increase in elevation, and the Spearman’s correlation coefficient between the two factors was r = -0.912 (P < 0.01), indicating a significant negative correlation. Thus, beautiful leisure villages were widely distributed at low elevations, and topographic factors were important indicators affecting the spatial distribution of beautiful leisure villages.

#### 3.6.2 Demographic factors

Based on the population statistics of China of 2020 and considering each province as a unit, China’s population density can be divided into five levels. Visualization of this data revealed high population densities in the southeast, which was correlated with the presence of a strong relationship between population and the presence of leisure villages. The number of beautiful leisure villages increased with the increase in the regional population, and the Spearman’s correlation coefficient between the two factors was r = -0.607 (P < 0.01), showing a significant positive correlation.

#### 3.6.3 Economic factors

The level of economic development is another important factor affecting the cultivation and spatial layout of beautiful leisure villages. The construction and development of beautiful leisure villages requires the support of a strong regional economy. Thus, when we use the per capita GDP of China’s provincial administrative regions from 2020 as the index to evaluate the degree of regional economic development, we could divide China’s economic development into five levels. Visualizations of this data revealed that China’s economically developed regions are primarily distributed across the southeastern coastal areas, while the areas with denser distribution of beautiful leisure villages were located in the Pearl River Delta, where both demonstrate a high degree of parity. The number of beautiful leisure villages increased with the increase in the per capita GDP, and the Spearman’s correlation coefficient between the two factors was r = -0.693 (P < 0.01), showing a significant positive correlation. Thus, more economically developed areas were likely to be characterized by more favorable location conditions, increased resources, and better infrastructure and service facilities, making it easier to promote innovation and gain broader acceptance of novel systems and ideas and helping to implement locally constructive beautiful leisure villages.

#### 3.6.4 Location factors

When we considered the distance between beautiful leisure villages and cities above the prefecture level as the measurement index for location factors, and used the natural fracture point to divide them into five levels, the distance between most beautiful leisure villages and cities above the prefecture level was < 80 km. Additionally, the driving distance between these beautiful leisure villages and larger cities was < 1 h,. This indicated that the distribution of these beautiful leisure villages largely depended on the urban layout. The closer it is to the city, the more it will be populated and frequented by residents of the city and more tourism resources will be collected. These centers also receive more support in terms of talent, technology, capital, and information. The number of beautiful leisure villages increased with the increase in per capita GDP, and the Spearman’s correlation coefficient between the two factors was r = -0.693 (P < 0.01), showing a significant positive correlation. Therefore, location factors play an important role in the formation and development of beautiful leisure villages and are important factors that affect the spatial distribution of leisure villages. Moreover, most beautiful leisure villages that were relatively far away from the city benefited from cultural tourism. Thus, the tourist market for these beautiful leisure villages was not limited to the surrounding urban population; rather, it attracted people from far distances by relying on their unique tourism resources.

## 4. Conclusion and discussions

### 4.1 Conclusion

Beautiful leisure villages across China demonstrated a clear spatial aggregation, with the spatial distribution at the provincial scale being particularly uneven. Despite this, this imbalance tended to weaken gradually as the scope of the evaluation moves up or down. On evaluating the distribution of these villages using cross-provincial economic zones, a largely unbalanced distribution was observed, revealing a clear trend of centralized distribution with the degree of aggregation initially increasing and then decreasing. The average accessibility time for these beautiful leisure villages was 197.24 min, and nearly 60% of all beautiful leisure villages had an accessibility time of > 2 h. Distribution differences in accessibility across the country were obvious, with obvious traffic directivity. The accessibility of beautiful leisure rural counties showed a strong agglomeration pattern, and the distribution of accessibility hot spots showed an obvious belt distribution from east to west. The distribution of these villages was also affected by the degree of economic development and rural resource endowment, with the service scope of these beautiful leisure villages moving from West>East> Middle. This distribution also clearly mirrored accessibility. In addition, topography, population, economy, and location were all crucial factors affecting the spatial distribution of beautiful leisure villages across China.

The spatial structure showed that North China, Southeast China, Southwest China, and other relevant provinces were the gathering areas of beautiful leisure villages in China. The formation of this spatial structure was closely related to the historical evolution of the countryside. First, rural settlements are related to agriculture. From animal husbandry and agricultural development to the improvement of agricultural production level, and from the formation of scattered settlements to the formation of fixed villages all consider the geographical environment. Among them, natural environmental factors are the basis for the formation and distribution of rural settlements. Natural factors, such as hydrology, topography and climate, affect agricultural production and the formation of villages. In North China, the terrain is flat with many arable lands. In the southwest and southeast regions, there are many mountains and hills, rivers, and sufficient water sources. These areas are favorable for cultivation, which is promotes the formation of villages. Conversely, economic, social, cultural, and other human environmental factors also have a certain impact on the formation of different types of beautiful leisure villages. New modern villages with relatively developed economy are generally distributed in the surrounding areas of cities. Areas inhabited by minorities are generally distributed with characteristic folk customs and residential villages. Old remote areas far from cities, with conventional transportation and beautiful environment, are generally distributed with historical villages. Second, irrespective of rural tourism demonstration sites, national agricultural tourism demonstration sites, or beautiful leisure rural sites, the development of the eastern, central, and western regions was uneven. The eastern regions were more developed than the central and western regions, which were closely related not only to the pattern of rural tourism development, but also to the imbalance of national policy guidance and economic development. The research results have certain theoretical significance and serve as a reference for the construction and planning of beautiful leisure villages and the protection of ecological environment.

### 4.2 Discussions

This paper analyzed the spatial distribution and current characteristics of various beautiful leisure villages on a macro scale and revealed the primary factors affecting their distribution, all of which have practical implications for the design and development of these beautiful leisure villages and the overall revitalization of the rural areas across China. The exemplary leading role of beautiful leisure villages helps to promote the organic integration of tourism industry and related industries, meet the requirements of tourists for rural tourism, and promote high-quality development of leisure rural tourism to realize symbiosis, win-win situations, and sustainability of leisure rural tourism and economic development. Despite these valuable contributions, there are still deficiencies in this paper. These include that the construction of a beautiful leisure countryside is only a single link in a larger program designed to reduce poverty and support rural revitalization, implying that its broader connections to other factors and the underlying mechanisms governing these systems need further evaluation. Despite the vast interest in the topic, coordinated research into the design and development of these leisure tourism villages and their connection with the local natural environment, social economy, tourism market, and other factors across different regions of China remains weak. This implies that there is a lack of meaningful understanding around the development conditions, market potential, and risk prediction and analysis for these projects. Thus, both identification and risk stratification measures require significant improvements. Given this, we recommend further in-depth evaluations with a more collective approach in the future to develop better construction plans and consequently, realize the true potential of these leisure village projects. Moreover, the construction and development of beautiful leisure villages should follow the principle of “green ecology and sustainable development,” consider building a beautiful country as the long-term goal according to their own characteristics, create rural types with their own characteristics, meet the requirements of tourists, expand farmers’ employment, and promote the sustainability of rural area development and construction.

## Supporting information

S1 Data (XLSX)
